# Nucleosome assembly and disassembly pathways *in vitro*

**DOI:** 10.1371/journal.pone.0267382

**Published:** 2022-07-13

**Authors:** Akiko Hatakeyama, Yuliia Shymko, Brigitte Hartmann, Romain Retureau, Claude Nogues, Marco Pasi, Malcolm Buckle

**Affiliations:** 1 RIKEN Center for Biosystems Dynamics Research, Chuo-ku, Kobe, Japan; 2 LBPA, UMR8113 CNRS, ENS Paris-Saclay, Université Paris-Saclay, Gif-sur-Yvette, France; Saint George’s University, UNITED KINGDOM

## Abstract

Structural fluctuations of nucleosomes modulate the access to internal DNA in eukaryotic cells; clearly characterisation of this fundamental process is crucial to understanding gene regulation. Here we apply PhAST (Photochemical Analysis of Structural Transitions) to monitor at a base pair level, structural alterations induced all along the DNA upon histone binding or release. By offering the first reliable, detailed comparison of nucleosome assembly and disassembly *in vitro*, we reveal similarities and differences between the two processes. We identify multiple, sequential intermediate states characterised by specific PhAST signals whose localisation and amplitude reflect asymmetries of DNA/histone interactions with respect to the nucleosome pseudo dyad. These asymmetries involve not only the DNA extremities but also regions close to the pseudo dyad. Localisations of asymmetries develop in a consistent manner during both assembly and disassembly processes; they primarily reflect the DNA sequence effect on the efficiency of DNA-histone binding. More unexpectedly, the amplitude component of PhAST signals not only evolves as a function of intermediate states but does so differently between assembly and disassembly pathways. Our observation of differences between assembly and disassembly opens up new avenues to define the role of the DNA sequence in processes underlying the regulation of gene expression. Overall, we provide new insights into how the intrinsic properties of DNA are integrated into a holistic mechanism that controls chromatin structure.

## Introduction

The fundamental repeating unit of chromatin is the nucleosome; 145–147 base pairs (bp) of DNA wrapped around an octamer of histone proteins (two H2A/H2B heterodimers and one (H3/H4)_2_ tetramer) [1, 2] that efficiently compacts genomes into cell nuclei and regulates many DNA functions [3, 4]. The spatial and temporal distribution of nucleosomes as a result of nucleosome assembly and disassembly is involved in all DNA transactions. *In vivo*, a plethora of interplaying factors such as chaperones, remodelling complexes, histone variants, epigenetic modifications and intrinsic, sequence dependent, DNA properties [5] orchestrate the dynamics of nucleosome positioning. A detailed knowledge of the biophysical basis underlying the structural pathways involved in nucleosome assembly and disassembly is a prerequisite to understanding such an important and fundamental cellular event as nucleosome biogenesis and turnover.

In recent years a number of studies have been conducted on the mechanism of nucleosome disassembly. Studies were carried out *in vitro* by recording the response of preformed nucleosomes to a gradual increase in ionic strength, using in particular the positioning DNA called the 601 or Widom sequence ([6] and references therein). Fluorescence-based techniques such as FRET (Förster Resonance Energy Transfer) were the preferred tools used to reveal the stepwise disassembly of nucleosomes, with the caveat that the fluorophores, bulky and hydrophobic, could perturb histone and nucleosome structures [7, 8]. These approaches, that typically used three or four pairs of fluorophores, provided data about specific histone-histone and histone-DNA interactions [7, 9–18]. Less frequently, an external mechanical force was applied to the DNA in a nucleosome [19–22]. The examination of spontaneous disassembly focused on unwrapping of DNA in the peripheral regions (so-called “DNA breathing”) [23–28].

It is now accepted that the global pathway for nucleosome disassembly involves the sequential release of histones or groups of histones. The series of FRET studies cited above as well as TR-SAXS (Time-Resolved Small Angle X-ray Scattering) [10, 28, 29] approaches proposed a pathway with two major successive phases: an initial release of the H2A/H2B dimers followed by (H3/H4)_2_-DNA dissociation. Such a global two-phase scheme is likely to be general since it was observed in nucleosomes studied under identical conditions but containing different DNA sequences, *i*.*e*., 601- and 5S-nucleosomes [15, 29] or 601-, 5S- and MMTV-nucleosomes [13, 17].

Although technically not straightforward, the existence of very early states of disassembly, before the removal of H2A/H2B, was also examined. With the 5S-nucleosome, the two H2A/H2B dimers were observed to dissociate from the (H3/H4)_2_-DNA complex in a single transition without observable stable intermediates [9, 29]. FRET experiments with 601-nucleosomes proposed that disruption or weakening of the interface between H2A/H2B dimers and the (H3/H4)_2_ tetramer (the so-called “butterfly” state), helped to rupture the DNA-H2A/H2B interfaces [12, 13, 16]. According to models inferred from experiments using SAXS [29], FRET [20] and single molecule unwrapping associated with FRET [20], the release of the two H2A/H2B dimers in 601-nucleosomes is asymmetric, starting from one unwrapped DNA end (the so-called “J”-shaped state). Two recent cryo-EM studies captured structures related to first events of spontaneous disassembly of 601-nucleosomes [26, 28] which are hard to observe in solution because nucleosome open states are marginally populated [23, 24]. The cryo-EM structures showed again an asymmetric loss of contacts between H2A/H2B and DNA arising from the spontaneous breathing of one extremity of the DNA fragment; this first nucleosome opening gradually propagates in association with subtle histone rearrangements. The intermediate states, in which one H2A/H2B dimer is no longer visible, resemble those hexasomes (DNA bound to the (H3/H4)_2_ tetramer and one H2A/H2B dimer) obtained from SAXS [10, 30] or X-ray crystallography [31]; the asymmetric opening was considered symptomatic of a DNA sequence effect since the strict symmetry of the histone structured domains with respect to the pseudo dyad axis [1, 32] cannot account for such phenomena. On the basis of salt titrations [32] and single molecule experiments [19] it was proposed that the 601 sequence is constituted by “strong” left and “weak” right halves [10, 33]; strong and weak sides may relate to differences in DNA intrinsic flexibility [34].

A general DNA sequence effect on disassembly was further attested by the fact that 601-nucleosomes better resist chaotropic destabilisation than nucleosomes formed with other sequences [15, 33]. In contrast, nucleosome stability, characterised by the ionic strength at the midpoint of the disassembly transition, is not affected by the histone origin [13] probably because of the very high degree of conservation of histone sequence and folding [35].

**Fig 1:**
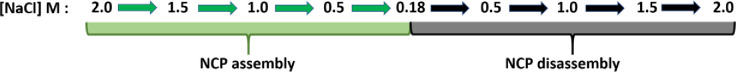
Outline of the PhAST experiments monitoring successive DNA structural changes induced during nucleosome assembly and disassembly by using stepwise decrease and increase of NaCl concentration.

The large number of studies presented above provided information heavily biased towards nucleosome disassembly; states preceding H2A/H2B dimer release are more completely characterised than later events leading to the complete dissociation of the complex. By comparison, with some notable exceptions [12, 36, 37], assembly has been poorly studied, thus limiting our understanding of nucleosome association and dissociation. In view of this, we embarked on studies to characterise nucleosome intermediate states during NaCl-induced assembly and disassembly (Fig 1) of the 601-nucleosome and to compare the two pathways. We used the PhAST (Photochemical Analysis of Structural Transitions) technique developed in our group [38].

PhAST allows precise (base pair resolution) quantification of positions on DNA where structural changes are induced by, for example, interactions with proteins: it is thus a powerful tool to detect DNA sequence effects which remain otherwise difficult to characterise even for image processing techniques [28]. PhAST is applicable to freely diffusing macromolecules in solution, and does not therefore require the use for example of fluorophores with their associated restrictions [7, 8]. PhAST measures the probability of forming UV photo-induced cyclobutane dimers between adjacent pyrimidines (YpY dimer, linked by C5-C5 and C6-C6 bonds) on the same DNA strand [38–40]. In addition to the quantum yields specific to each type of step, the probability of forming YpY dimers depends on the local YpY structure; more precisely on roll and twist, two inter base-pair parameters that are coupled in B-DNA in solution [41]. Thus, low twist and positive roll shorten the YpY C5-C5 and C6-C6 distances and thus favour dimer formation whereas large twist and negative roll have the inverse effect [38]. The YpY dimer-formation probabilities along a DNA sequence therefore reflect its local average structure. Comparison between probabilities collected on free and bound DNA simultaneously reveals DNA structural changes induced by the presence of proteins.

PhAST proved to be remarkably efficient in following structural changes in DNA as 601- and derived 601-nucleosomes were formed under decreasing ionic strength conditions, *i*.*e*., during nucleosome assembly [38], as summarised now. As expected, nucleosome formation starts in the central regions of the 601 sequence where both copies of (H3/H4)_2_ bind and terminates at the 5’ and 3’ ends with the recruitment of H2A/H2B dimers. The intuitive idea that histone binding induces noticeable structural changes in the local parameters of roll and twist [6] all along the 601 sequence in the final nucleosome structure was demonstrated. An original result was the detection and description of intermediates occurring during nucleosome formation. The structural organisation of these nucleosome intermediate states reflected the existence of marked DNA sequence effects that could be unambiguously assigned to specific DNA regions. For example, (H3/H4)_2_ interacts more robustly with the 5’ side of the 70 bp central segment of the 601 sequence than with its 3’ side counterpart, an asymmetry that is not present when the 5’ side is mutated at key points. These results were explained by the experimental asymmetries found in the dynamic properties of the free sequence [34]. That such subtle events could be captured by PhAST encouraged us to apply it to 601-nucleosome disassembly allowing us to compare both the assembly and disassembly processes.

In this manuscript we first examined the potential effect of salt concentration to induce structural perturbations in free DNA, by examining PhAST data collected on the free 601 sequence at various ionic strengths and comparing the YpY reactivities. Having ascertained that there was no detectable alteration in DNA structure even at 2M NaCl, any differences in YpY reactivities between free and bound DNA could be interpreted in terms of strengthened or weakened DNA/histone interactions (ultimately, histone binding or release). The DNA/histone interactions were defined from previous molecular simulations in explicit solvent that provided a fine mapping of contacts between DNA and not only the structured histone regions but also their unstructured tails [42]. The dual approach of combining structural changes in DNA during nucleosome turnover with known DNA/histone interactions in the final nucleosome allowed us to describe a series of major intermediate states that appear during both assembly and disassembly processes. These intermediates clearly respond to the sequence dependent properties of free DNA; however, a given ionic strength does not induce the same intermediates during assembly and disassembly, a fact that may have critical biological implications for nucleosome turnover.

## Materials and methods

### DNA sequence

The 601 sequence of 147 bp is given in S1 Table indicating that which we refer to here as the 5’- and 3’ halves. This sequence was previously used in the context of the experimental study of nucleosome assembly [38] and for exhaustive all atoms simulations in explicit solvent [42, 43].

### Assembly

DNA fragments containing the 601 sequence were prepared as described previously [38] and reconstituted using the salt dilution method according to the manufacturer’s instructions (New England BioLabs) with a slight modification [38]. Human recombinant histone H2A/H2B dimer (5.5 μg, 203 pmol) and histone (H3/H4)_2_ tetramer (5.5 μg, 102 pmol) (New England BioLabs) were mixed with linearised 601 DNA fragments (10 μg, 23 pmol) in 30 μl of 2M salt buffer (18 mM Tris-HCl, 2 M NaCl, 0.9 mM DTT, 0.9 mM EDTA). The mixture was incubated at room temperature (RT) for 30 min before the salt concentration was lowered to 0.18 M by adding dilution buffer (10 mM Tris-Cl, pH 7.5, 1 mM EDTA, 0.05% NP-40, 5 mM 2-mercaptoethanol, 0.1 mM PMSF) five times every 20 min (from 2 M to 1.5 M, 1.0 M, 0.5 M and 0.18M NaCl). Samples were removed from the stock solution at every step of dilution following 20 min incubation and mixed rapidly with an equivalent NaCl concentration solution to a final sample volume of 20 μl (DNA concentration was 50 ng/μl for all conditions; H2A/H2B histone dimer concentration was 27.5 ng/μl, (H3/H4)_2_ tetramer concentration was 27 ng/μl). Samples were kept on ice for a minimum of 20 minutes before laser irradiation. Free DNA was treated similarly in parallel for further data processing. Micrococcal nuclease (MNase) digestion previously performed at 0.1 M NaCl produced footprints that confirmed that the nucleosomes are homogenous and stable at this ionic strength [38].

### Disassembly

Nucleosome complexes (and free DNA) were formed as described above and concentrated using an Amicon Ultracel-10 Centrifugal Filter (Millipore). Solutions at 0.18 M NaCl were transferred directly to either 0.5 M, 1.0 M, 1.5 M or 2M NaCl, the DNA and histone concentrations were maintained at 50 ng/μl, 27.5 ng/μl (H2A/H2B histone dimer), and 27 ng/μl ((H3/H4)_2_ tetramer). Solutions were incubated for a minimum of 20 min before laser irradiation. As for assembly, free DNA was treated similarly in parallel for further data processing. The incubation times here lie within the spectrum of times used in other disassembly studies [10, 11, 16, 33], and are compatible with those used in assembly to facilitate comparison.

### PhAST

#### Technique

20 μl of nucleosomes at each step of assembly/disassembly were irradiated with 5-ns-long pulses of 266nm UV laser beam at a frequency of 10Hz for a period of 1sec (Quanta-Ray INDI-40-10 10 pulsed Nd: YAG). After irradiation, DNA fragments were purified by phenol-chloroform extraction and ethanol precipitation followed by primer extension using Taq polymerase (New England BioLabs). Two primers (A and B) with 6-FAM labelling at the 5’ end, were used to analyse the sites of the UV photoproducts of the 601 sequences (Primer: 5′-GCT-ATG-ACC- ATG-ATT-ACG-CCA-AGC-3′, Primer B: 5′- AGG-GTT-TTC-CCA-GTC-ACG-ACG-TT- 3′). For primer extension, 200-500ng of the UV irradiated DNA were mixed in 20μl of the final volume of an amplification mixture (Taq polymerase (0.5U), dNTPs (0.2mM), Taq polymerase buffer (1×), each primer (0.02mM) and distilled water). Primer extension consisted of denaturation at 95°C for 6min, primer annealing at 55°C for 1min, and extension at 72°C for 9min was carried out. The products were then concentrated by ethanol precipitation. All samples were re-suspended in a 10μl solution containing 9.75μl deionized formamide and 0.25μl GeneScan-600 LIZ internal size standard (Applied Biosystems). The mixture was denatured for 5min at 95°C and separated by capillary electrophoresis using a 3500 genetic analyser (Applied Biosystems).

#### Analysis of capillary electrophoretograms

**Fig 2:**
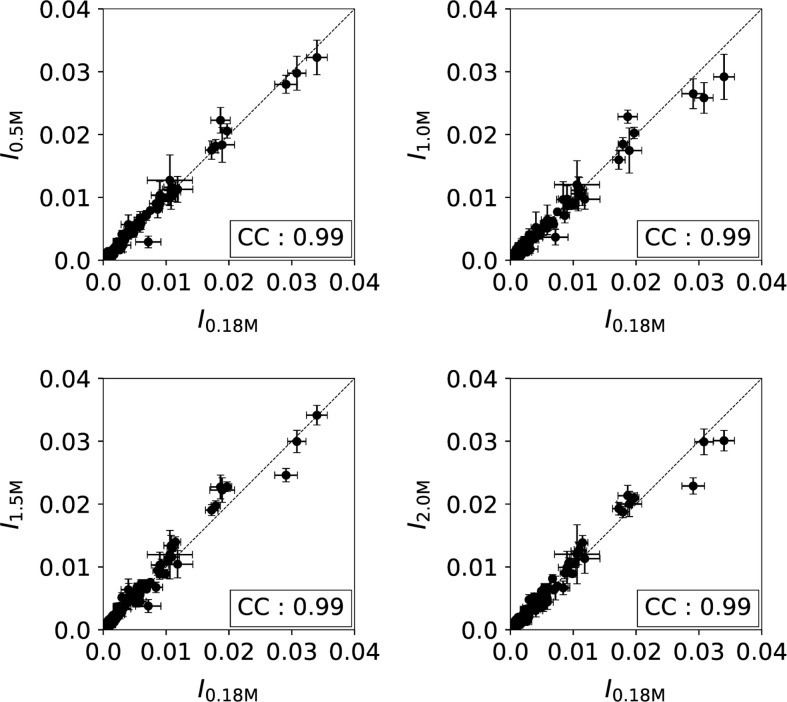
Comparison of PhAST signals in free DNA at various ionic strengths. The Phast signal is expressed in terms of intensity (I) that is the normalised peak height associated with each YpY step of the free 601 sequence. The data collected at 0.5, 1.0, 1.5 and 2 M NaCl were systematically compared to those at 0.18 M NaCl. Each point represents the value of I, averaged from 6 experiments related to assembly and disassembly studies; the vertical and horizontal bars are standard errors. The correlation coefficients (CC) are given in boxes in each panel.

The resulting capillary electrophoretograms (for an example, see S1 Fig and Fig 2 of our previous publication [38]) were analysed to determine the size (number of nucleotides) and relative abundance of the fragments present in each sample, according to the following procedure. First the electrophoretogram was calibrated by converting migration times to fragment sizes (in units of nucleotides) through piecewise linear fits to the internal size standard (600LIZ) which was run together with each sample. The initial part of each electrophoretogram (up to and including 20 nucleotides) consistently displayed extremely noisy behaviour and was systematically discarded from further analyses. Note that since the primers used for primer extension were larger than 20 nucleotides (see above), the retained portion of electrophoretograms also contained the unelongated primers. To facilitate comparison among independent CE runs, electrophoretograms were then normalised using their integral. Peaks were identified based on the analysis of the numerical first derivative of the electrophoretograms, and their maximum height was taken as an estimation of the relative abundance of each fragment. Knowing that a fragment of length *x* indicated the formation of a pyrimidine dimer between nucleotides *x*+1 and *x*+2, to obtain the relative propensity of forming a pyrimidine dimer at each position along our DNA, we needed to assign to each peak an integer fragment size in units of nucleotides. The starting point for this assignment was the calibrated migration time corresponding to the peak maximum, which was, by the very nature of the calibration process, a fractional quantity in units of nucleotides. Instead of simply taking the closest integer size by rounding, which often leads to artefacts such as assigning two peaks to the same fragment size, we developed an optimization procedure that minimised artefacts by allowing small corrections (on average of about 0.24 nucleotides in either direction), rigorously without changing the order of peaks. In particular, we took advantage of the fact that the size of a fragment implies its sequence, and made the reasonable assumption that the most likely cause for the polymerase to stop is the presence of a photo-induced pyrimidine dimer. Our procedure maximizes, within the aforementioned constraints, the likelihood of the fragment length assignment given its sequence. The resulting sets of peak intensity as a function of DNA sequence for *n* independent replicates at the same salt concentration were averaged (*n* = 3, 4 or 5 depending on the condition), and the standard error was calculated.

#### Quantification of induced DNA structural changes

To quantify changes in the likelihood of pyrimidine dimer formation due to histone binding at a given salt concentration, we calculated the ratio between the peak intensity in the sample with DNA and histones (D+H) over the sample with DNA alone (D) for each pyrimidine dinucleotide on both strands. These intensity ratios (IR) indicate how much more or less likely it is for a given pyrimidine dimer to form in the presence of histones. As described previously [38], the comparison is best presented using the log_2_ of these intensity ratios (log_2_(*IR*)); in particular, in the following we represent absolute values (|log_2_(*IR*)|, see e.g., Fig 4) in order to simplify the comparison of the effects of histone binding along the sequence. The standard error σ for the log_2_(*IR*) values was estimated using the following formula:

$${\left(\ln\;2\right)}^{2}\sigma^{2}={\left(\frac{\sigma_{D+H}}{\mu_{D+H}}\right)}^{2}+{\left(\frac{\sigma_{D}}{\mu_{D}}\right)}^{2}$$

propagating the standard error calculated for each condition (*σ*_*D*+*H*_ and *σ_D_* refer to samples in the presence or absence of histones, respectively) using a first order expansion around their means (*μ*_*D*+*H*_ and *μ_D_*).

Since salt concentration doesn’t affect by itself the behaviour of DNA (see Results and Fig 2), we assume that the observed differences can only be ascribed to the effect of histone binding, and in particular of DNA wrapping around the histone core. We therefore set out to study the behaviour of regions of the DNA of various sizes, such as entire helical turns or regions that will be in contact with different histones in the fully formed nucleosome core particle (NCP, for more details on the definition of these regions see below). To do so we sum the |log_2_(*IR*)| values over each DNA region: the resulting values are indicative of the local (within each DNA region) difference in behaviour between DNA in presence of histones versus DNA alone, i.e., they quantify the effect of histone binding on the local structure of DNA. It is interesting to note that this is equivalent to considering each DNA region as a point in multi-dimensional space where each coordinate is the (log_2_) intensity of a pyrimidine dinucleotide (YY) PhAST signal, and measuring the structural distance between the DNA in presence of histones and DNA alone (as we are summing absolute values along multiple dimensions, this is a Manhattan distance). Furthermore, this procedure also strengthens the statistical significance of our comparisons. Since the number of YYs per region varies depending on the local DNA sequence, these distances are better represented by normalising their value by their dimensionality.

#### Interpretation of induced DNA structural changes

In order to provide a structural interpretation of our data, we take the 2.0 M NaCl trace to be the baseline: since histones don’t bind to DNA at this concentration, the effect of histone binding on the local structure of DNA, as measured by the distances defined above, is at its minimum. We compare distances measured at each salt concentration with the corresponding 2.0 M NaCl value using Student’s t-test. Distances that are not significantly different from the 2.0 M NaCl value indicate that the behaviour of DNA is indistinguishable from that of free DNA, that is the region is either not bound to histones, or it is bound but the DNA is unmodified by the histones. In either case, it seems reasonable to consider that the region cannot be wrapped around the histone core, and we therefore assign these regions to be “unwrapped”. On the other hand, when the distances are significantly different (p < 0.05), under our assumptions (see above) we assign the region to be “wrapped”. Within this category, we further compared the distances with the corresponding values at 0.18 M, which correspond to the behaviour of the fully formed nucleosome core particle (NCP). When the behaviour is significantly different from that of the NCP (p < 0.01), we have termed these as "intermediate" states, whose behaviour is significantly different from both free DNA and the fully formed NCP.

### DNA/histone interface analysis

The DNA/histone interface was defined from previously published molecular simulations of nucleosome models containing the 601 sequence, which were performed in explicit solvent [42, 43]. One advantage of these models was that they contained large parts of histone tails that are not resolved in crystallographic structures. The contacts between DNA and both structured and unstructured histone regions, described in detail in an article specifically devoted to this topic [42], were analysed by VLDM (Voronoi Laguerre Delaunay for Macromolecules) as polygonal surfaces, quantified by their area and occurrence, without resorting to any empirical or adjusted parameters. In the present paper, those contacts occurring less than 20% of the simulation time were not considered. This criterium was in fact sufficient to also eliminate very small contact areas, less than 2 Å^2^.

Note that the DNA sequence is expressed in terms of Super Helical Location (SHL) that is, the number of helical turns separating a given base pair from the central base pair, SHL0; we assume that, on average, one turn corresponds to 10 bp.

## Results

PhAST generates YpY dimers in DNA using laser photo-radiation; the dimer detection technique produces peaks representing the probabilities of dimer formation at each YpY position along a sequence (S1 Fig); the quantification consists of measuring the peak amplitudes which we will call intensities (*I*). The intensities (*I*) reflect the DNA local structure, as reported in the Introduction. Broadly speaking, the PhAST experiments followed the outline shown in Schema 1, using either decreasing or increasing ionic strengths to study assembly or disassembly, respectively. At the highest (2 M NaCl) and lowest (0.18 M NaCl) ionic strengths, intensities (*I*) correspond to free DNA or DNA fully engaged in a nucleoprotein complex, respectively. By comparing data collected throughout the different stages of nucleosome assembly and disassembly with data collected on free DNA photo-irradiated in parallel, PhAST analysis described events characterising the assembly and disassembly pathways by revealing the structural effects of protein binding or release on DNA.

Before presenting our analysis of differences in PhAST signals associated with variations in salt concentration and their interpretation in terms of changes in DNA/histone interactions, we focus on free DNA to determine whether ionic strength variations in the range used here perturbed neither its structure nor the quantum yield of YpY dimer formation.

### PhAST profiles of free DNA at various NaCl concentrations

Our studies of nucleosome assembly and disassembly used salt concentrations from 0.18 to 2.0 M NaCl. Variations within this range modify the melting temperature of free B-DNA [44] without strongly perturbing the gyration radius, persistence length [45–47] or intrinsic torsional stiffness [48]. Also, the force associated with unzipping of the double helix remains almost unchanged from 10 mM to 1 M NaCl [49]. However, to our knowledge, there is no report on possible effects of ionic strength variations on the local structure of the double helix at room temperature. We therefore decided to examine the effects of salt concentration on the PhAST signals of free DNA to obviate any potential bias on interpretation of the data comparing free and bound DNA. The PhAST signals collected for each position along the free 601 sequence at 0.5, 1.0, 1.5 or 2.0 M NaCl were therefore compared to those obtained at 0.18 M NaCl (Fig 2). In all cases, the four pairs of normalised intensities (0.5 vs 0.18 M, 1 vs 0.18 M, etc…) were highly correlated and aligned according to x = y (CC = 0.99, Fig 2). This clear result showed that over the considered range, the salt concentration had a marginal effect on the quantum yield of YpY dimer formation and on the average local DNA structure.

We remarked however that two specific YpY steps were extremely sensitive to laser photo-radiation regardless of the ionic strength (Fig 2, intensity (*I*) > 0.03). These “hotspots” correspond to TpT dinucleotides immediately 5’ TpA in the two TTTAA segments present in the 601 sequence. According to an NMR study [50] the free TTTAA oligomer is associated with low twists and positive rolls that both should favour YpY dimer formation [38]. In addition, the two adenines facing the photo-reactive TpT step show uncommon behaviour that includes an exceptional sensitivity of their ^31^P chemical shifts to temperature changes [50] and the presence of slow motions [51]. The enhanced photo-reactivities associated with these steps provides a further example of the ability of PhAST to detect unusual structural features in DNA.

In the context of the present work, differences between PhAST signals collected at various ionic strengths can be confidently interpreted in terms of modifications of DNA structure due to DNA/histone interactions. The next requirement is to have a precise description of the DNA/histone interface in solution.

### DNA/histone interface

**Fig 3:**
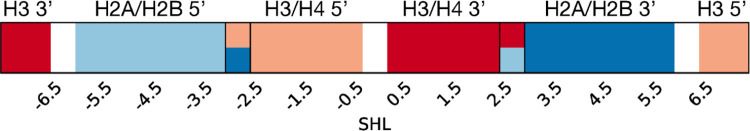
DNA regions involved in the DNA/histone interface. This schematic representation shows those DNA regions that interact with the different histone dimers in the fully formed NCP. To distinguish the two copies of the H2A/H2B dimer, they are labelled 5’ (light blue, chains G, H) and 3’ (dark blue, chains C, D). Similarly, the two pairs of H3/H4 histones are labelled 5’ (pink, chains E, F) and 3’ (red, chains A, B). The DNA sequence is labelled as SHL (Super Helical Location, defined in Material and Methods). The denominations A, B, … of the histone chains are those commonly used in X-ray structures.

A 1.2 μs molecular dynamics trajectory in explicit solvent was recently obtained on a nucleosome containing the 601 sequence and histones with large portions of the tails [42]. Monitoring of the DNA-protein contacts with VLDM, a program specifically devoted to analyse such interactions, allowed very fine documentation of the interface [42]. Here, we revisited these data to focus on those DNA regions in which nucleotides of one or the other strand of the double helix interact with the histones H3, H4, H2A or H2B (details in S2 Fig, summary in Fig 3).

Overall, the contact area associated with (H3/H4)_2_ tetramers represent 54% of the total contact area, thus slightly larger than for H2A/H2B heterodimers. The DNA/histone interactions are largely symmetrical with respect to the pseudo dyad axis (Fig 3 and S2 Fig) and thus parallel the strict symmetry of the histone structured domains; this implies that the DNA sequence, which is not palindromic, has a marginal effect on the interface once the DNA is fully wrapped around the histone and a complete nucleosome is formed.

Three short DNA fragments, one just 5’ of the pseudo dyad and two others located before the extremities are not in contact with the histones. Furthermore, differentiating between the effects induced by the binding of the (H3/H4)_2_ tetramer or the H2A/H2B dimers is facilitated by the fact that DNA regions where interactions with H3/H4 and H2A/H2B overlap are limited (Fig 3).

### Assembly and disassembly processes

PhAST was applied to samples of DNA alone (D) and DNA plus histones (D+H) at five ionic strengths for both assembly and disassembly, according to Schema 1. Capillary electrophoresis following primer extension after irradiation gave traces such as those shown in S1 Fig. Structural differences between free and bound or partially bound DNA were monitored and quantified by attributing to each YpY position an absolute value of log_2_ of Intensity Ratios, defined as |log_2_(*IR*)| = absolute value of the log_2_ of the average normalised intensity of a given peak in the D+H samples divided by the average normalised intensity of the same peak in the D samples (for more details, see Materials and Methods). This quantity measures the structural distance between the DNA in the D+H sample at a given ionic strength and free DNA taken as reference; |log_2_(*IR*)| has an expected value of zero when the DNA in the D+H sample is not bound to the histones. It was reassuring to observe that PhAST data from assembly experiments obtained here were largely compatible with those obtained previously [38] (S3 Fig).

**Fig 4:**
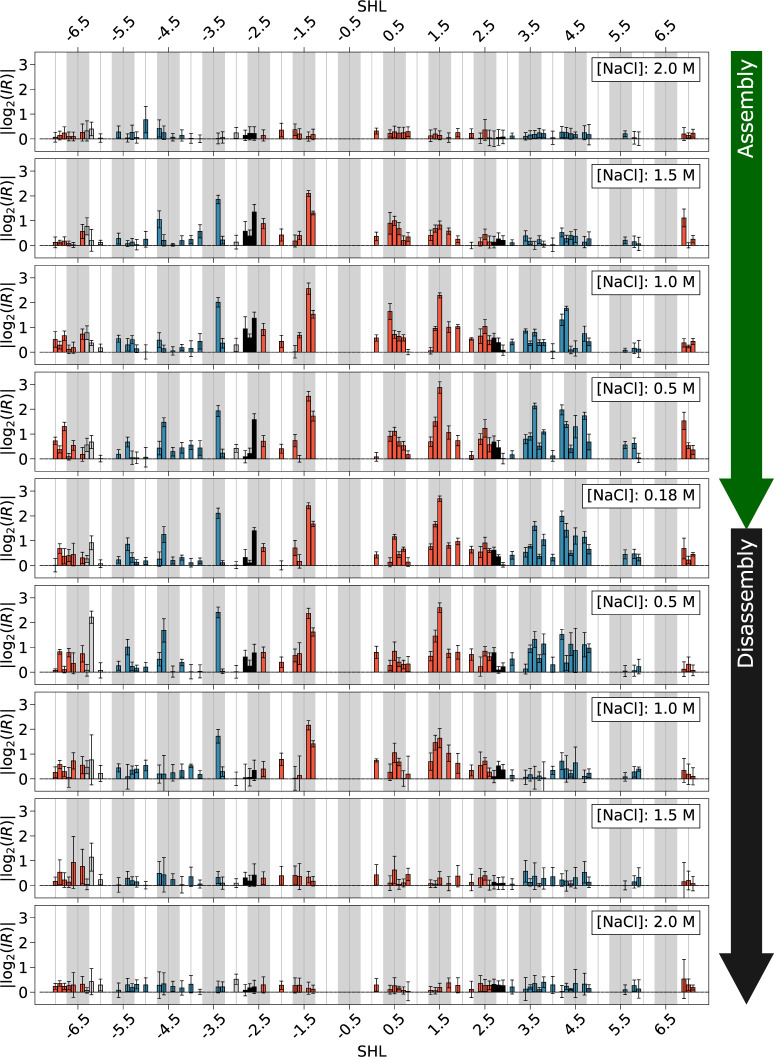
Changes in the probability of YpY dimer formation in DNA during nucleosome assembly and disassembly experiments. Changes in the probability of YpY dimer formation are presented in terms of absolute values of log2 of the intensity ratios (|log2(IR)|) along the 601 sequence expressed in SHLs; they are given for decreasing (top panel) or increasing (bottom panel) ionic strengths, as indicated by the green and black arrow respectively. The IR quantities are the ratios calculated between the normalised peak heights associated with each YpY step in the histone plus DNA mixtures and those of DNA alone. Red and blue bars correspond to DNA residues involved in the interface with H3/H4 and H2A/H2B dimers, respectively; black bars correspond to dinucleotides contacted by the two dimers. Minor-groove inward facing regions observed in the nucleosome structures are represented by grey boxes. Error bars are first-order estimations of standard errors calculated on at least 3 independent experiments (see Material and Methods for details).

The profiles inferred from data collected during assembly and disassembly experiments (Fig 4) show that most YpY steps are associated with significant |log_2_(*IR*)| values between 0.18 and 1.5 M NaCl. At 0.18 M NaCl, the DNA was involved in a stable, complete nucleosome as confirmed by micrococcal nuclease footprinting. Thus, the corresponding |log_2_(*IR*)| distance profile represents a signature of the structural effect of the histone octamer on the DNA in a fully formed nucleosome. At 2.0 M NaCl, |log_2_(*IR*)| values are very weak, reflecting the large predominance of free DNA. Note that these values are averages affected by noise and, for this reason, can never be zero as ideally expected for free DNA. Finally, we note that |log_2_(*IR*)| values at the peripheral SHL’s ± 6.5 are often small in amplitude and associated with large standard errors (Fig 4), which may severely compromise a concrete interpretation. This could reflect a large range of conformations including, even at 0.18 M NaCl, a proportion of DNA that is free from histones. Indeed, the DNA in the SHL extremities is known to undergo breathing. [9, 11, 24, 26, 52] Such dynamic events seem to be accentuated at the 3’ extremity [28]. These terminal regions (up to SHL ± 6) are therefore excluded from the following analyses.

### Differences between assembly and disassembly pathways

**Fig 5:**
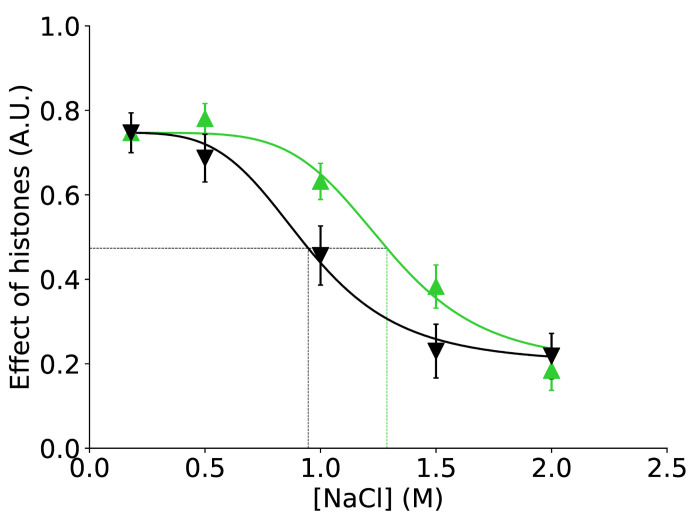
Effect of histones on DNA structure as calculated by summing |log_2_(IR)| values over the whole 601 sequence (excluding up to SHL ± 6). The data associated with different stages of assembly (green triangles) and disassembly (black inverted-triangles) are compared at each ionic strength; values are divided by the total number of pyrimidine dinucleotides. Error bars are first-order estimations of standard errors; differences at 1.0 M and 1.5 M are statistically significant (p < 0.05, Student’s t-test). Sigmoidal fits to the data (black and green curves) are used to estimate the approximate concentration of NaCl at which 50% of the range of total change is attained (black horizontal line) for assembly (dotted green vertical line) and disassembly (dotted black vertical line).

Fig 4 shows that the patterns of |log_2_(*IR*)| values are not strictly the same in assembly and disassembly. To gain a global view over these differences, we summed all values along the 601 sequence at each step of the assembly and disassembly process. The resulting distances (see also Material and Methods) are indicative of the difference in behaviour between DNA in the presence of histones versus DNA alone, i.e., they quantify the effect of histone binding on the structure of DNA (Fig 5).

Fig 5 clearly demonstrates the huge differences observed between the assembly and disassembly processes. For both assembly (green curve) and disassembly (black curve), decreasing salt concentration increases the effect of histones on the structure of DNA, as DNA incrementally wraps (or unwraps) the histones: the effect is minimal at 2.0 M (but not 0, see above) and maximal at 0.18 M, as expected. During assembly 50% of the final effect of histones are already observed at around 1.3 M NaCl whereas during disassembly 50% this point is seen at around 0.9 M NaCl (note that the ordinate is not normalised and that these are absolute values).

To more clearly understand successive events occurring during assembly and disassembly experiments, we sum |log_2_(IR)| distances over the DNA regions that contact the different histone dimers in the complete nucleosome, as defined in Fig 3 and S2 Table. In passing, we remark that the effect of histone binding on the local structure of the H2A/H2B 5’ region of DNA is rather small even in the fully formed NCP: a possible explanation for this is that nucleosome wrapping of this DNA region has a small average effect on its structure.

**Fig 6:**
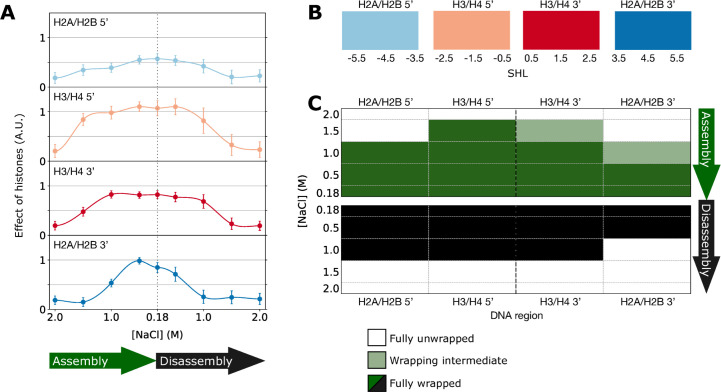
Effect of histones on DNA regions interacting with histone dimers in the fully wrapped NCP. **A**: Effect of histones as calculated by summing |log2(IR)| over the DNA regions interacting with the histone dimers in the complete nucleosome. Error bars are 1.5 times standard error, such that no overlap between the error bars indicates significant differences (with circa p < 0.05). **B:** schematic representation of the DNA regions that are associated with the histone dimers in the complete nucleosome. **C**: Statistical analysis of the traces in **A** interpreted as the behaviour of each region at each salt concentration, classified as either fully unwrapped, fully wrapped or intermediate (see Materials and Methods). The ordinate shows salt concentration and the abscissa is detailed in **B**. The top half of the figure (green) refers to assembly and the lower part (black) to disassembly.

The result of this analysis, shown in Fig 6, confirms the striking feature that assembly and disassembly pathways are not equivalent, and reveals how the various regions of DNA contribute to the overall behaviour observed in Fig 5. At 1.0 and 1.5 M NaCl, assembly/disassembly differences emerge very clearly over the DNA-H2A/H2B 3’ region (dark blue in Fig 6) but also over the central regions, where H3/H4 interact (pink and red in Fig 6). The most spectacular divergence between association and dissociation paths occurs at 1.5 M NaCl: during association contacts between the H3/H4 tetramer and DNA are well established (although those involving H2A/H2B are not in place) whereas during dissociation all histone/DNA interactions are absent.

### Nucleosome assembly and disassembly: A structural model

In an attempt to relate the PhAST data to potential structures present at the various intermediate salt concentrations, we classify each DNA region according to whether its behaviour is more similar to that of unwrapped, linear DNA, or of the fully wrapped NCP (respectively white and green/black in Fig 6C). In cases where the behaviour is significantly different from both, we call these regions intermediates (light green in Fig 6C; for more details see Materials and Methods). It is worth noting that, although each of these three behaviours may be the result of an equilibrium of multiple structures, this third group may especially be indicative of the coexistence of wrapped and unwrapped DNA. A point supporting this approach is that the structures of bound and free DNA regions in partially wrapped intermediates of nucleosome disassembly (e.g., hexasomes and tetrasomes) closely resemble those observed within the fully wrapped canonical nucleosomes and free DNA, respectively [30, 31, 53].

Fig 6C recapitulates the assembly and disassembly pathways as suggested by our PhAST approach. In agreement with previous observations [38], assembly begins at 1.5 M NaCl by forming an initial complex in which the DNA region immediately 5’ of the pseudo dyad is fully wrapped, while the corresponding 3’ region isn’t yet but its structure is nevertheless affected by the wrapping, compatible with the formation of an asymmetric tetrasome-like structure. At 1.0 M NaCl, an asymmetric nucleosome-like structure develops around a now symmetric and fully formed DNA-(H3/H4)_2_ tetrasome. At 0.5 M NaCl, the DNA is experimentally indistinguishable from its structure in the fully formed nucleosome, in both assembly and disassembly. According to our data, unwrapping is clearly taking place between 0.5 and 1.0 M NaCl with the loss of the structural effect in the H2A/H2B 3’ region; at 1.0 M NaCl our data are compatible with the formation of a hexasome. At 1.5 M NaCl, our data are consistent with full unwrapping: one can consider that the DNA is not associated with histones. This pathway is in line with previous disassembly studies [11, 16] that reported stable nucleosomes up to around 0.6 M NaCl, a transition towards the hexasome centred at 0.55 M NaCl, followed by total dissociation via a tetrasome above about 1.25 M NaCl [11, 16]. This correspondence also suggests that the incubation times used here, although shorter than times used in some other disassembly studies, [11, 12, 16] are sufficient such that the equilibrium we observe is similar to that described by others, at least during disassembly.

Despite differences, both assembly and disassembly of the two DNA sides occur asymmetrically with respect to the DNA pseudo dyad axis: the whole right (3’) side of the 601 sequence appearing to be less competent in creating or maintaining the wrapped conformation than the left (5’) counterpart. As shown in Fig 6, during assembly, at 1.5 M NaCl the H3/H4 5’ region is fully wrapped, whereas the H3/H4 3’ region is not. Similarly, the H2A/H2B 5’ region is fully wrapped at 1.0 M NaCl, whereas the H2A/B 3’ region is not. Unwrapping of the H2A/H2B regions is even more clearly asymmetric during dissociation; at 1.0 M NaCl, the H2A/H2B 3’ region is fully unwrapped whereas the H2A/H2B 5’ regions is fully wrapped. It is impossible to attribute asymmetry to disassembly for the H3/H4 regions, as at 1.5 M NaCl DNA is fully unwrapped.

## Discussion

The main aim of this study was to provide a comparison of nucleosome formation and dissociation pathways under the same conditions. We used the PhAST approach that is non-invasive and does not require the use of chemical modifications. The unambiguous detection of intermediate, partially wrapped states in terms of location along the whole nucleosome DNA is one of the major advantages of PhAST to investigate transitions in nucleosome organisation especially with regard to following both assembly and disassembly.

**Fig 7:**
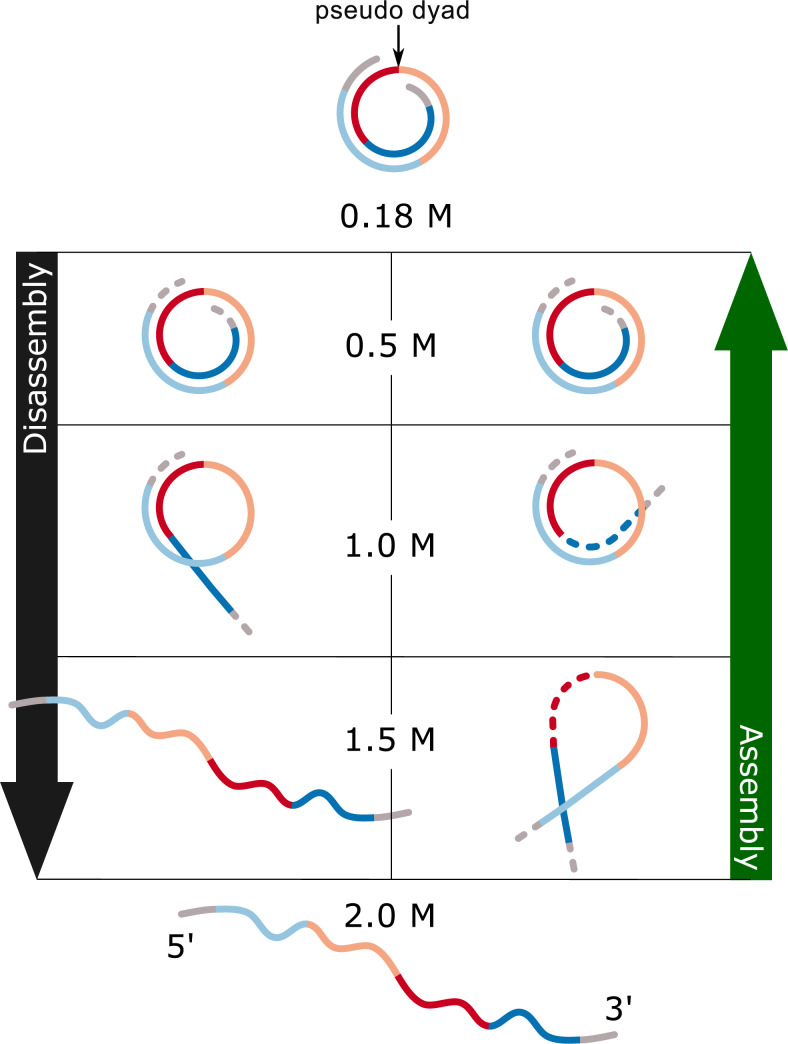
A structural model of putative intermediates during nucleosome turnover in vitro. The model shows a cartoon of proposed structures present at different stages of assembly (green arrow) and disassembly (black arrow). The 601 sequence is represented as a four-colour line that corresponds to DNA regions that are associated with the histone dimers in the complete nucleosome with regard to the pseudo dyad axis (see Fig 3 for details). The upper schematic represents the organisation of the SHL’s on the complete nucleosome as a left-handed helix going into the page from the 5’ end.

Clearly, multiple states are formed sequentially during both nucleosome assembly and disassembly where the DNA is only partially wrapped around the histone core. Our approach allows the construction of structural models of the partially wrapped states as a function of salt concentration (Fig 7), based on the statistical analysis of our data (Fig 6). Our PhAST approach confirms the paradigm that during assembly, wrapping takes place from the DNA pseudo dyad and progresses towards the extremities whereas during disassembly, unwrapping develops in the direction from the extremities to the centre. The disassembly pathway we observe is consistent with previous results obtained using independent techniques [11, 16]; interestingly, our data are compatible with the existence of ‘asymmetric open’ and ’teardrop’ structures described under similar conditions [10] at 1.2 M NaCl during disassembly.

In addition, PhAST reveals that the directionally inverse progressions of DNA wrapping/unwrapping are not strictly symmetrical with respect to the pseudo dyad axis: the 5’ side (pink and light blue in Fig 7) is more favourable for wrapping than the 3’ side (red and dark blue in Fig 7) in both assembly and disassembly pathways, confirming and extending previous observations obtained for disassembly [11, 16, 33]. This asymmetrical behaviour with respect to the pseudo dyad axis may reflect the DNA sequence effects on the ability to wrap around the histones. According to what is predicted from the TRX scale that quantifies the intrinsic structural variability in B-DNA at the dinucleotide level [34]), the 601 sequence consists of asymmetric halves. Globally, the properties of the 5’ half coincide much better than those of the 3’ half with the periodic variations of the structure of the nucleosomal DNA; the 5’ half is therefore thought to limit the cost of DNA wrapping [34, 50]. We suggest that the disassembly pathway exploits the most stressed regions in a manner analogous to the relaxation of a stiff spring upon release of constraints. Indeed, our work indicates that those DNA regions that are particularly refractive to nucleosome formation are also the first to break their interactions with the histones.

However, by far the most striking results of our study is the difference observed between assembly and disassembly (Figs 5–7). In simple terms, different partially wrapped states exist under different conditions depending on whether one is assembling from free histones and DNA or disassembling from reconstituted nucleosomes, themselves formed by the assembly process. This apparent ’hysteresis’ is a novel observation that is not an artefact of experimental conditions since care was taken to maintain ionic strength and reagent concentrations equal during both assembly and disassembly. There may be several explanations for this heterogeneity between assembly and disassembly. In the absence of data to the contrary we offer the following explanation. During assembly, asymmetric contacts at 1.5 M NaCl between exposed hydrophobic regions on H3/H4 stabilise an initial tetramer-DNA interaction. Subsequent wrapping of the DNA, recruitment of H2A/H2B and associated distortions severely disrupt this hydrophobic interaction which is compensated by electrostatic interactions and the important free energy gain associated with wrapping—especially left-handed wrapping of right-handed DNA. During dissociation, the pre-existing nucleosome does not expose these hydrophobic sites on H3/H4 such that the structures resulting from unwrapping at increasing ionic strength do not have the compensatory stabilising effect of hydrophobic interaction formation. This suggest a form of histone folding that is correlated with concerted binding to DNA as a result of decreasing ionic strength. The reverse unfolding of histones upon release from the DNA may not follow the same pathway and not expose hydrophobic regions for binding to DNA; put another way, the DNA is no longer necessary to provide a suitable hydrophobic environment since the histones have adopted a separate configuration.

This is of course extremely speculative. As a model it does however present some predictive capacity. Histone proteins, due to their high positive charge, are liable to spurious aggregation, they are therefore necessarily associated with specific chaperones in order to maintain some form of structural integrity within the cell [54–57]. Consequently, although *in vitro* ionic strength manipulation conditions most likely are not to be found *in vivo*, we assume that nucleosome turnover *in vivo* is similar to that observed here. For example, *in vivo* H3/H4 histones ’released’ to the DNA from chaperones would undergo a similar type of transition that we observe during assembly at 1.5 M NaCl.

Based on the hysteresis-like behaviour observed here, we suggest that disassembly is more cooperative than assembly; the idea of just inverted transitions is therefore too simplistic. Further *in vitro* support for this model comes from a previous study that had as its main objective the measurement of rates of spontaneous unwrapping and rewrapping and to measure the kinetics of protein binding site exposure [52]. These FRET data showed that disassembly is faster than assembly. Furthermore, a recent simulation study showed that the degree of cooperativity of disassembly was affected by external conditions [58]. Collectively, these observations may explain why chromatin disruption in front of the replication fork is faster than reorganisation behind the fork. This lag is postulated to provide an opportunity for targeted transcription factor binding to the newly replicated DNA [59].

In summary, the PhAST approach allowed us to obtain snapshots of intermediates in a continuous process of gradual rearrangements during nucleosome turnover, showing that the assembly and disassembly pathways do not correspond to strictly invertible schemes. Our detailed analysis provides unambiguous evidence for the role of the DNA sequence in determining the relative stability of these intermediates, through its effect on local DNA structure and stiffness. We suggest that the DNA sequence contributes to nucleosome positioning mainly through differential stabilisation of these intermediates, rather than of fully formed particles. The ability to reliably investigate these processes makes PhAST a useful tool for the study of how DNA sequence affects chromatin remodelling *in vivo*, and creates exciting opportunities to design DNA sequences with varying affinities for histones that could be useful to fully explore the complex relationship between DNA sequence and nucleosome positioning. It seems reasonable to expect that such DNA sequence effects modulate chromatin remodelling in conjunction with the numerous trans factors such as ATP-dependent proteins that regulate binding, release and sliding of nucleosomes.

## Supporting information

S1 Fig Capillary electrophoretograms at 0.18 M NaCl and 1.5 M NaCl. Capillary electrophoresis after photo-irradiation and primer extension of 601 fragments; red lines show DNA alone, blue lines show DNA in the presence of histones. Representative electrophoretograms for one strand and one replica per condition are shown (for a total of 8 conditions). Asterisks indicate the positions of YpY steps along the sequence. Panels 1A and 2A show data from assembly, 1B and 2B show data from disassembly.
(PDF)

S2 Fig DNA-histone contacts from nucleosome models. These plots show which DNA regions interact with the different chains (A, B, C, etc…) of each histone type (H3-A and H4-B in light and dark red, H2A-C and H2A-D in grey and black, H3-E and H4-F in light and dark purple, H2A-G and H2A-H in light and dark blue). The contacts are characterised by their areas, calculated for each base pair along the DNA. The DNA sequence is labelled by SHL (Super Helical Location, defined in Material and Methods). These DNA/histone interface data were extracted and analysed from nucleosome simulations [42].
(PNG)

S3 Fig Comparison between PhAST data from this work and from previous investigations. log_2_(*IR*) values were collected at 0.5, 1.0 and 1.5 M NaCl (total of 190 points, correlation coefficient of 0.82) during assembly experiments carried out for this work or for previous investigations [38].
(PNG)

S1 Table The 601 sequence. (DOCX)

S2 Table DNA regions that bind histone dimers and number of YpY steps. (DOCX)
