# Supplementary figures and images for: Nucleosome assembly and disassembly pathways in vitro
Source: PLoS One. 2022 Jul 13;17(7):e0267382. doi: 10.1371/journal.pone.0267382 (PMC9278766; doi:10.1371/journal.pone.0267382)

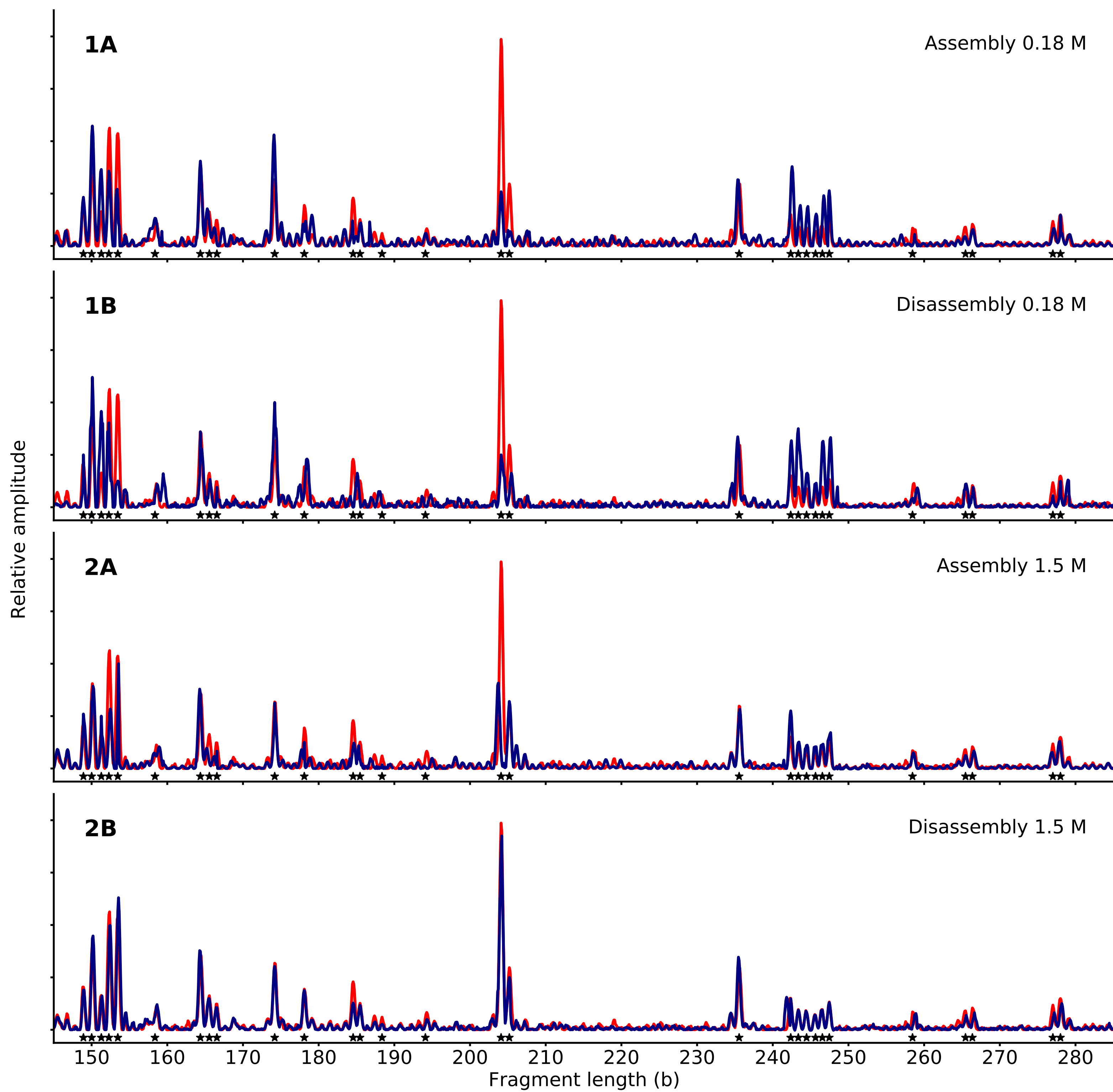

Supplement: S1 Fig — Capillary electrophoresis after photo-irradiation and primer extension of 601 fragments; red lines show DNA alone, blue lines show DNA in the presence of histones. Representative electrophoretograms for one strand and one replica per condition are shown (for a total of 8 conditions). Asterisks indicate the positions of YpY steps along the sequence. Panels 1A and 2A show data from assembly, 1B and 2B show data from disassembly. (PDF) [file pone.0267382.s001.pdf]

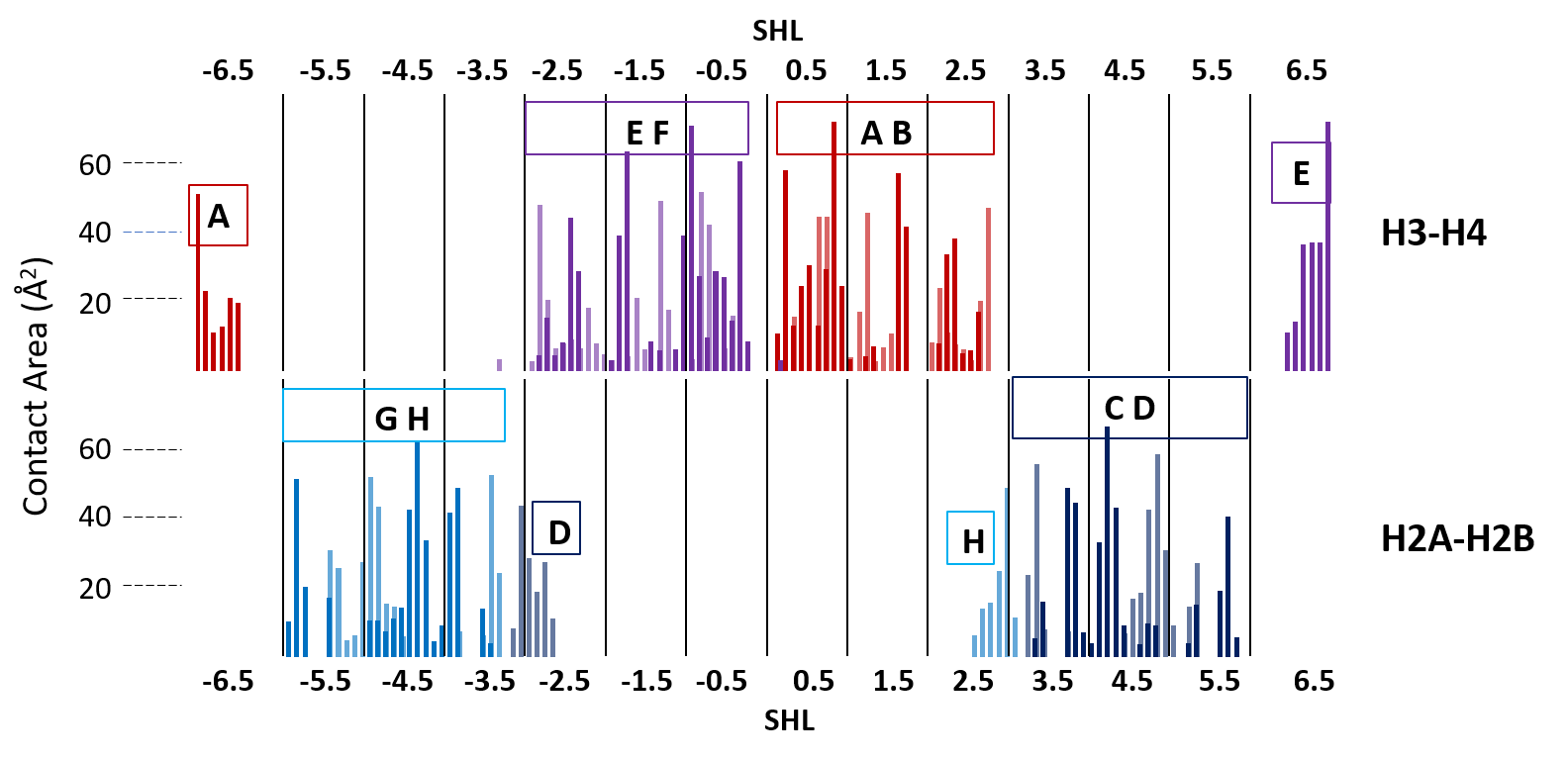

Supplement: S2 Fig — These plots show which DNA regions interact with the different chains (A, B, C, etc…) of each histone type (H3-A and H4-B in light and dark red, H2A-C and H2A-D in grey and black, H3-E and H4-F in light and dark purple, H2A-G and H2A-H in light and dark blue). The contacts are characterised by their areas, calculated for each base pair along the DNA. The DNA sequence is labelled by SHL (Super Helical Location, defined in Material and Methods). These DNA/histone interface data were extracted and analysed from nucleosome simulations [42]. (PNG) [file pone.0267382.s002.png]

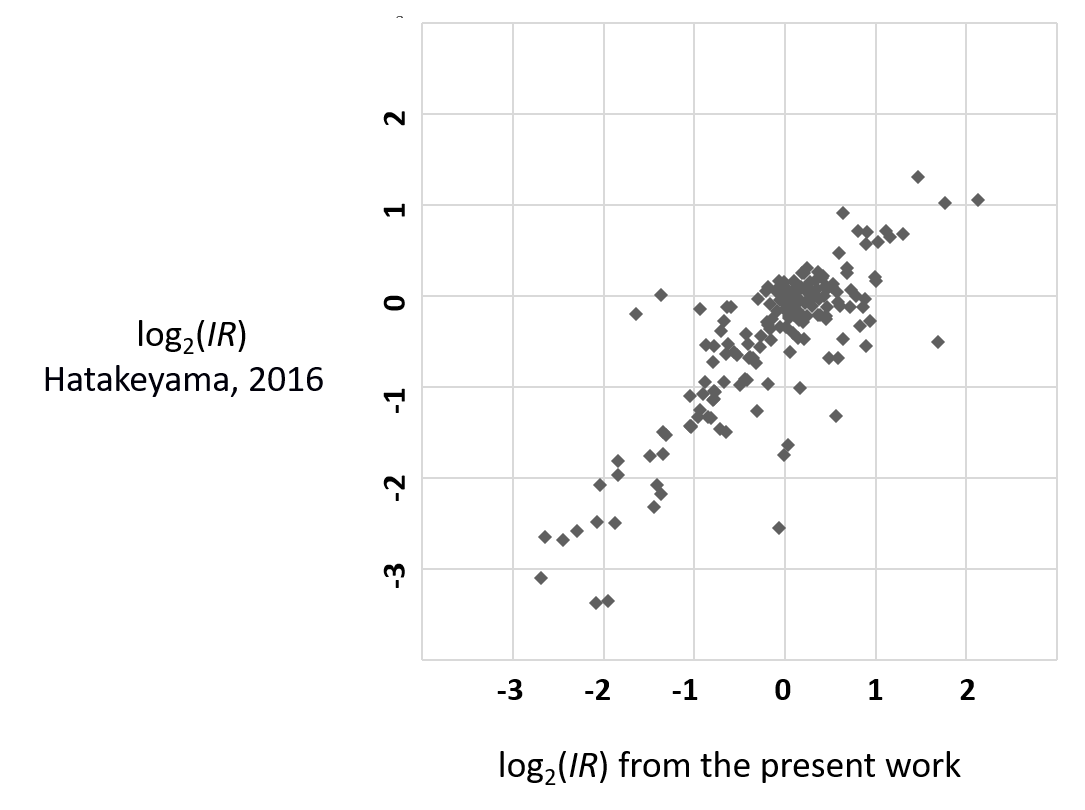

Supplement: S3 Fig — log2(IR) values were collected at 0.5, 1.0 and 1.5 M NaCl (total of 190 points, correlation coefficient of 0.82) during assembly experiments carried out for this work or for previous investigations [38]. (PNG) [file pone.0267382.s003.png]
