# Supplementary material for: Nucleosome assembly and disassembly pathways in vitro
Source: PLoS One. 2022 Jul 13;17(7):e0267382. doi: 10.1371/journal.pone.0267382 (PMC9278766; doi:10.1371/journal.pone.0267382)
Supplement: S1 Table — (DOCX) [file pone.0267382.s004.docx]

**S1 Table. The 601 sequence.** This table specifies the sequence of one strand of the so-called 601 sequence, in which SHL ±1.5 regions are underlined and the sequence centre is indicated by a cyan background. 601 5’ and 3’ halves are also detailed in terms of percentages of G•C or A•T base pairs.

| Sequence 601  147 bp | 5'-CTGGAGAATCCCGGTGCCGAGGCCGCTCAATTGGTCGTAGACAGCTCTAGCACCGCTTAAACGCACGTACGCGCTGTCCCCCGCGTTTTAACCGCCAAGGGGATTACTCCCTAGTCTCCAGGCACGTGTCAGATATATACATCCTGT  -3' |
| --- | --- |
| 5’ half  73 bp  60% of G•C bp | 5'-CTGGAGAATCCCGGTGCCGAGGCCGCTCAATTGGTCGTAGACAGCTCTAGCACCGCTTAAACGCACGTACGCG  -3' |
| 3’ half  73 bp  48% of A•T bp | 5'-TGTCCCCCGCGTTTTAACCGCCAAGGGGATTACTCCCTAGTCTCCAGGCACGTGTCAGATATATACATCCTGT  -3' |
