# Supplementary material for: Nucleosome assembly and disassembly pathways in vitro
Source: PLoS One. 2022 Jul 13;17(7):e0267382. doi: 10.1371/journal.pone.0267382 (PMC9278766; doi:10.1371/journal.pone.0267382)
Supplement: S2 Table — (DOCX) [file pone.0267382.s005.docx]

**S2 Table. DNA regions that bind histone dimers and number of YpY steps.** This table specifies the first (N1) and last (N2) nucleotides that are chosen here as the boundaries of the DNA regions that bind histone dimers in the complete nucleosome. The number of corresponding YpY steps is given in the last column.

| Histone dimer | N1 | N2 | n(YpY) |
| --- | --- | --- | --- |
| H3 A (tail) | -70 | -64 | 5 |
| H2A-H2B G-H | -59 | -33 | 13 |
| H3-H4 E-F | -25 | -1 | 6 |
| H3-H4 A-B | 3 | 26 | 14 |
| H2A-H2B C-D | 33 | 60 | 15 |
| H3 2 (tail) | 65 | 70 | 2 |
